# Assessment of Generative Artificial Intelligence (AI) Models in Creating Medical Illustrations for Various Corneal Transplant Procedures

**DOI:** 10.7759/cureus.67833

**Published:** 2024-08-26

**Authors:** Kayvon A Moin, Ayesha A Nasir, Dallas J Petroff, Bosten A Loveless, Omeed A Moshirfar, Phillip C Hoopes, Majid Moshirfar

**Affiliations:** 1 Hoopes Vision Research Center, Hoopes Vision, Draper, USA; 2 School of Medicine, American University of the Caribbean, Cupecoy, SXM; 3 Ophthalmology, University of Louisville School of Medicine, Louisville, USA; 4 Ophthalmology, Idaho College of Osteopathic Medicine, Meridian, USA; 5 Ophthalmology, Rocky Vista University College of Osteopathic Medicine, Ivins, USA; 6 Sam Fox School of Design and Visual Art, Washington University in St. Louis, St. Louis, USA; 7 John A. Moran Eye Center, University of Utah School of Medicine, Salt Lake City, USA; 8 Eye Banking and Corneal Transplantation, Utah Lions Eye Bank, Murray, USA

**Keywords:** eye, ophthalmology, pkp, dalk, dmek, dsaek, endothelial keratoplasty, medical illustration, image generation, artificial intelligence

## Abstract

Purpose: This study aimed to task and assess generative artificial intelligence (AI) models in creating medical illustrations for corneal transplant procedures such as Descemet's stripping automated endothelial keratoplasty (DSAEK), Descemet's membrane endothelial keratoplasty (DMEK), deep anterior lamellar keratoplasty (DALK), and penetrating keratoplasty (PKP).

Methods: Six engineered prompts were provided to Decoder-Only Autoregressive Language and Image Synthesis 3 (DALL-E 3) and Medical Illustration Manager (MIM) to guide these generative AI models in creating a final medical illustration for each of the four corneal transplant procedures. Control illustrations were created by the authors for each transplant technique for comparison. A grading system with five categories with a maximum score of 3 points each (15 points total) was designed to objectively assess AI's performance. Four independent reviewers analyzed and scored the final images produced by DALL-E 3 and MIM as well as the control illustrations. All AI-generated images and control illustrations were then provided to Chat Generative Pre-Trained Transformer-4o (ChatGPT-4o), which was tasked with grading each image with the grading system described above. All results were then tabulated and graphically depicted.

Results: The control illustration images received significantly higher scores than produced images from DALL-E 3 and MIM in legibility, anatomical realism and accuracy, procedural step accuracy, and lack of fictitious anatomy (p<0.001). For detail and clarity, the control illustrations and images produced by DALL-E 3 and MIM received statistically similar scores of 2.75±0.29, 2.19±0.24, and 2.50±0.29, respectively (p=0.0504). With regard to mean cumulative scores for each transplant procedure image, the control illustrations received a significantly higher score than DALL-E 3 and MIM (p<0.001). Additionally, the overall mean cumulative score for the control illustrations was significantly higher than DALL-E 3 and MIM (14.56±0.51 (97.1%), 4.38±1.2 (29.2%), and 5.63±1.82 (37.5%), respectively (p<0.001)). When assessing AI's grading performance, ChatGPT-4o scored the images produced by DALL-E 3 and MIM significantly higher than the average scores of the independent reviewers (DALL-E 3: 10.0±0.0 (66.6%) vs. 4.38±1.20 (29.2%), p<0.001; MIM: 10.0±0.0 (66.6%) vs. 5.63±1.82 (37.5%), p<0.001). However, mean scores for the control illustrations between ChatGPT-4o and the independent reviewers were comparable (15.0±0.0 (100%) vs. 14.56±0.13 (97.1%); p>0.05).

Conclusion: AI is an extremely powerful and efficient tool for many tasks, but it is currently limited in producing accurate medical illustrations for corneal transplant procedures. Further development is required for generative AI models to create medically sound and accurate illustrations for use in ophthalmology.

## Introduction

Corneal opacity is among the top five causes of blindness worldwide and can lead to severe visual impairment [1]. Many degenerative diseases like keratoconus and Fuchs dystrophy can negatively impact vision and necessitate corneal transplantation to restore a person's refractive power. Within the last several decades, corneal specialists have popularized not only full-thickness penetrating keratoplasty (PKP) but also partial lamellar corneal transplants such as Descemet's stripping automated endothelial keratoplasty (DSAEK), Descemet's membrane endothelial keratoplasty (DMEK), and deep anterior lamellar keratoplasty (DALK). The aforementioned surgical techniques have been well described in the literature [2-4]. Noting the novelty of such surgical approaches, it is important to clarify to both patients and clinicians the differences between these techniques. One way to educate the public in a succinct and coherent manner is through medical illustrations. 

Generative artificial intelligence (AI) models like Decoder-Only Autoregressive Language and Image Synthesis 3 (DALL-E 3) and Chat Generative Pre-Trained Transformer-4o (ChatGPT-4o) are at the forefront of AI advancements. Developed by OpenAI, DALL-E 3 generates images from textual descriptions, making it a powerful tool for creating detailed and contextually relevant illustrations across various fields [5]. Similarly, ChatGPT-4o, known for its human-like text generation, can also create AI-driven images with descriptions [6]. OpenAI enables developers to market and create custom GPTs for specific tasks, including medical illustrations. Together, these AI technologies demonstrate the potential to produce accurate and detailed visual and textual aids beneficial for medical education.

In this study, we aim to explore the capabilities of DALL-E 3 and Medical Illustration Master (MIM) [7] to determine their performance in generating medical illustrations for DSAEK, DMEK, DALK, and PKP using standardized, non-professionally engineered prompts.

## Materials and methods

Study design

This study adhered to the tenets of the Declaration of Helsinki, and the Hoopes Vision Ethics Committee and the Biomedical Research Alliance of New York (BRANY) Institutional Review Board (IRB) approved this study procedure (approval number: A20-12-547-823).

DALL-E 3 and MIM, two OpenAI platforms capable of creating medical illustrations, were chosen for our study. These platforms were found through a search of OpenAI's GPT store using the keywords "medical illustration."

**Figure 1:**
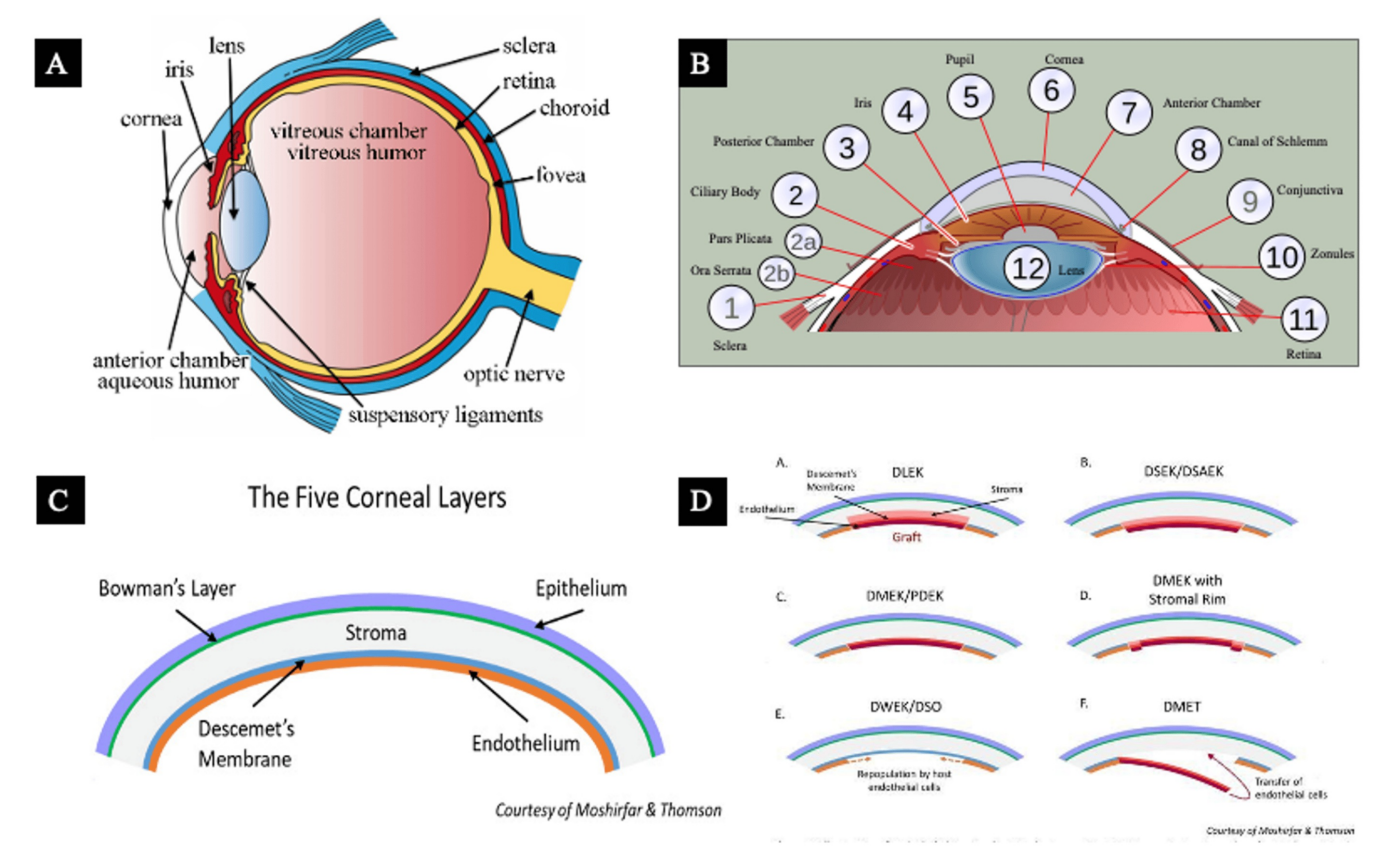
Example image for administered AI prompt number 5 Used for both DALL-E 3 and MIM showing the following: (a) ocular anatomy, (b) anatomy of the anterior segment, (c) cross-section of the cornea, and (d) basic procedural diagram of endothelial keratoplasty techniques Panels (a) and (b) have been adapted from an open-access source, courtesy of Dr. Holly Fischer [8] and Dr. Jordi Nogue [9], respectively. Panels (c) and (d) have been adapted from an open-source book [2] distributed under the terms of the Creative Commons Attribution-NonCommercial-NoDerivatives 4.0 International (CC BY-NC-ND 4.0) (https://creativecommons.org/licenses/by-nc-nd/4.0/) AI: artificial intelligence; DALL-E 3: Decoder-Only Autoregressive Language and Image Synthesis 3; MIM: Medical Illustration Master; DSAEK: Descemet's stripping automated endothelial keratoplasty; DMEK: Descemet's membrane endothelial keratoplasty; DALK: deep anterior lamellar keratoplasty; PKP: penetrating keratoplasty; DWEK: descemetorhexis without endothelial keratoplasty; DMET: Descemet's membrane endothelial transfer

Six prompts were then engineered for each of the four transplant procedures to allow the AI models to refine their produced images (Table 1). Example images created by the authors or found through a Google Images search were provided in prompt number 5 for each of the transplant procedures to aid the direction of the desired final illustration from these models (Figure 1A-1D [2,8,9]). Each set of six prompts was provided to the AI models in new conversations to mitigate the influence of previous interactions. The AI models generated images after each prompt (DALL-E 3: 48 total; MIM: 24 total), but only the final images produced were chosen for assessment (DALL-E 3: eight total; MIM: four total). It is important to note that DALL-E 3 created two images for each transplant procedure.

**Table 1:** Administered AI prompts with the corresponding explanation Six non-engineered prompts administered to both DALL-E 3 and MIM for each transplant procedure with its corresponding purpose AI: artificial intelligence; DALL-E 3: Decoder-Only Autoregressive Language and Image Synthesis 3; MIM: Medical Illustration Manager

Administered AI prompts	Purpose of prompt
1. Create a medical illustration for the corneal transplant procedure in question while ensuring the medical illustration is anatomically and surgically accurate.	1. Determine if AI is able to discern a medically accurate response and acknowledgment of prompt.
2. Depict all surgical steps in a sequential fashion, with each step portrayed onto one singular eye.	2. Evaluate programs' understanding of each surgical procedural step and ability to display it accurately in sequential order.
3. For each surgical step, include a brief caption summarizing each step and place it within the image.	3. Assess the ability of an AI model to recognize specific individual steps from an illustration, develop an accurate explanation that describes each step, and integrate captions seamlessly into the illustration.
4. Make each depicted iris purple while maintaining image clarity and legibility.	4. Test the AI programs' ability to recognize a specific part of ocular anatomy from a fabricated illustration, and then enact a modification on the said part.
5. Adjust the formatting to match the following example (see Figure 1A-1D).	5. Judge the ability of AI programs to interpret an uploaded image and modify its fabricated illustration to match the provided image template.
6. Condense the image to include only the three most pertinent steps of the procedure while retaining captions describing the relevant steps.	6. Gauge comprehension skills of the AI program, examining its ability to condense information while maintaining utility.

**Figure 2:**
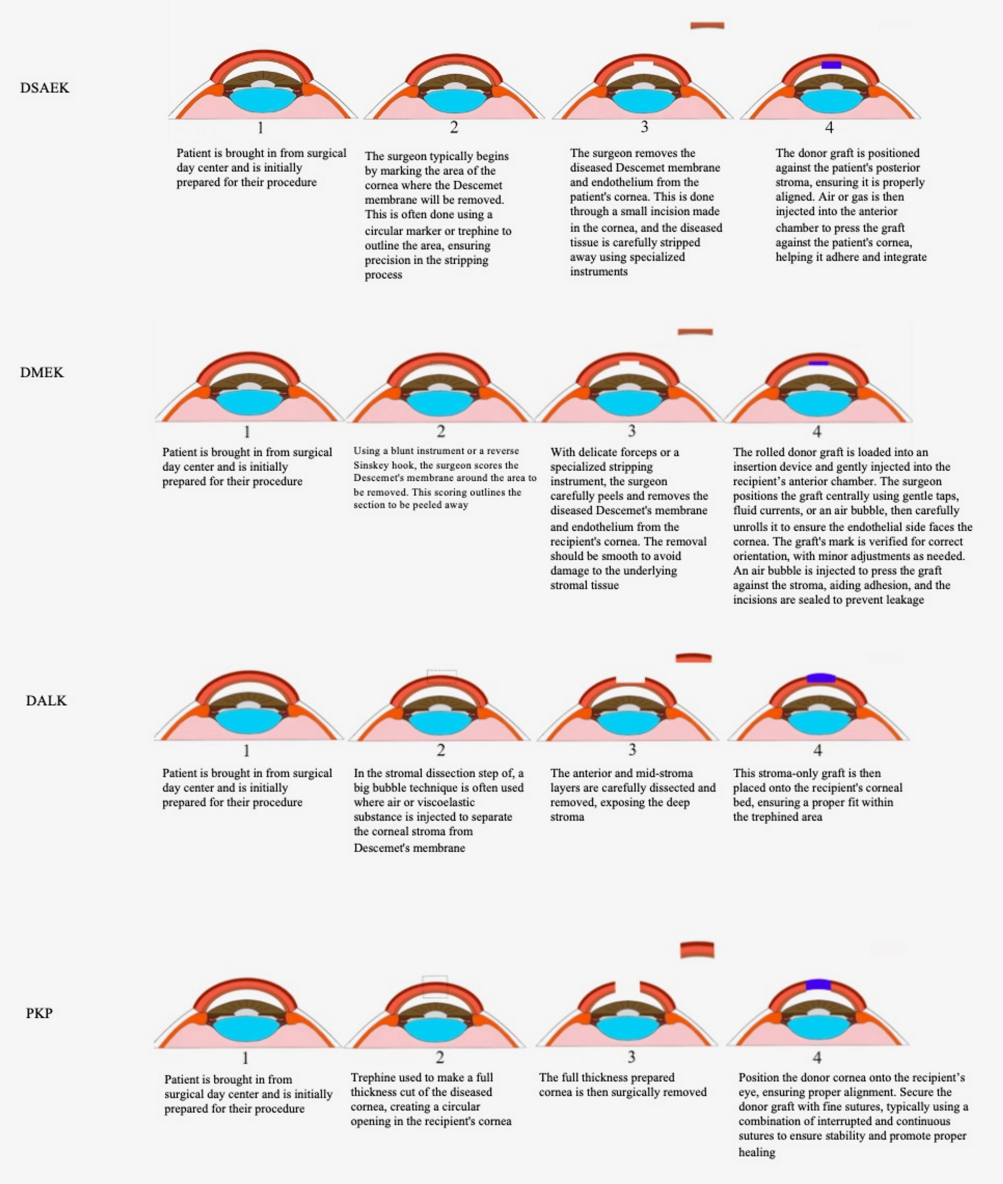
Control illustrations for DSAEK, DMEK, DALK, and PKP used as a comparison for the AI-generated images Image created by author A.A.N. using *Adobe Illustrator* AI: artificial intelligence; DALL-E 3: Decoder-Only Autoregressive Language and Image Synthesis 3; MIM: Medical Illustration Master; DSAEK: Descemet's stripping automated endothelial keratoplasty; DMEK: Descemet's membrane endothelial keratoplasty; DALK: deep anterior lamellar keratoplasty; PKP: penetrating keratoplasty

A control illustration for each of the four transplant procedures detailing accurate anatomy, appropriate procedural methodology, and detailed images was created by the authors to compare to the illustrations produced by the two AI models (Figure 2). A grading system with five categories was then designed to objectively assess all images: "Legibility," "Detail & Clarity," "Anatomical Realism & Accuracy," "Procedural Step Accuracy," and "Lack of Fictitious Anatomy." A description of each of these categories can be found in the Appendices. Each category had a maximum of 3 points, deeming a total possible score of 15 points per image/transplant procedure. Four independent reviewers graded the four transplant procedure images from both AI models as well as the control illustrations. All results were tabulated and graphically depicted.

In order to assess AI's grading capabilities, the four final images produced by DALL-E 3 and MIM as well as the control illustrations created by the authors were provided to ChatGPT-4o. ChatGPT-4o was then provided with the grading system described above to objectively score these images. These scores were then compared to those of the four independent reviewers.

Statistical analysis

Statistical analysis was conducted using Excel (Microsoft Corporation, Redmond, Washington, United States). Given our categorical independent variable and numerical dependent variable, one-factor analysis of variance (ANOVA) with post hoc Tukey testing was used to compare the mean numerical scores of the control illustrations, DALL-E 3, and MIM for all four transplant procedures. Welch's two-sample t-test for unequal standard errors was used to compare mean scores between reviewers and ChatGPT-4o for the assessment of AI's grading performance. A threshold of 0.05 was used to define statistical significance for reported observations. Levene's test was used to assess the equality of variances. The population's variances were considered to be equal (p=0.09). The normality assumption was assessed with the Shapiro-Wilk test (⍺=0.05). Due to the small sample size used for statistical analysis, the distributions were deemed to be not normal. However, the ANOVA test is considered robust for moderate violation of the normality assumption. Post hoc power analysis for mean cumulative scores demonstrated a statistical power of 0.30. 

## Results

**Figure 3:**
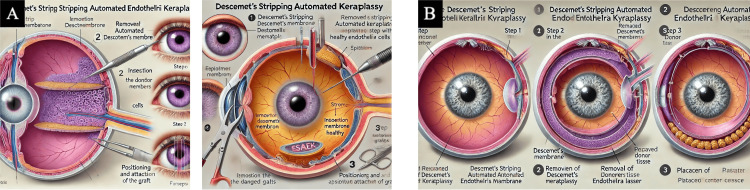
DSAEK DSAEK medical illustrations created by the generative AI models after inputting final prompt number 6 (Table 1). Panel (a) final image generated by DALL-E 3. Panel (b) final image generated by MIM AI: artificial intelligence; DALL-E 3: Decoder-Only Autoregressive Language and Image Synthesis 3; MIM: Medical Illustration Master; DSAEK: Descemet's stripping automated endothelial keratoplasty

**Figure 4:**
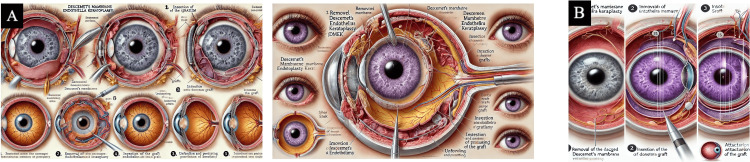
DMEK DMEK medical illustrations created by the generative AI models after inputting final prompt number 6 (Table 1). Panel (a) final image generated by DALL-E 3. Panel (b) final image generated by MIM AI: artificial intelligence; DALL-E 3: Decoder-Only Autoregressive Language and Image Synthesis 3; MIM: Medical Illustration Master; DMEK: Descemet's membrane endothelial keratoplasty

**Figure 5:**
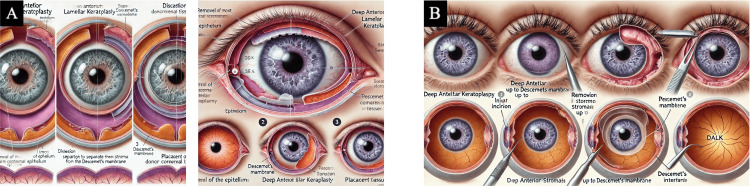
DALK DALK medical illustrations created by the generative AI models after inputting final prompt number 6 (Table 1). Panel (a) final image generated by DALL-E 3. Panel (b) final image generated by MIM AI: artificial intelligence; DALL-E 3: Decoder-Only Autoregressive Language and Image Synthesis 3; MIM: Medical Illustration Master; DALK: deep anterior lamellar keratoplasty

**Figure 6:**
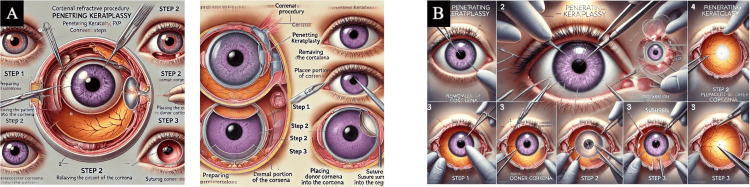
PKP PKP medical illustrations created by the generative AI models after inputting final prompt number 6 (Table 1). Panel (a) final image generated by DALL-E 3. Panel (b) final image generated by MIM AI: artificial intelligence; DALL-E 3: Decoder-Only Autoregressive Language and Image Synthesis 3; MIM: Medical Illustration Master; PKP: penetrating keratoplasty

See Figures 3-6 for the resultant AI images generated for each corneal transplant procedure.

Observational findings

Both AI models demonstrated strong language processing capabilities but struggled with accurate medical imagery. Despite the vibrant and artful nature of the generated diagrams, they were grossly inaccurate. An issue that occurred in 100% of the generated images was the creation of multiple irides or full eyes within an already existing eye. For procedural steps involving unique techniques, such as the "bubble" technique in DALK, the AI would create bubbles within the eye. As shown in Figure 3A in the DSAEK procedure, what appear to be cells of the cornea are inside the vitreous of the eye. Inappropriate anatomy appeared in 100% of the images, particularly in Figure 4A, which shows an expanded choroid, the inaccurate use of the optic nerve, and the creation of various accentuated vascular pathways. Additionally, the text generated in images was often misspelled, blurred, or illegible.

Individual category scoring

The maximum score in this section is 3 points. 

**Figure 7:**
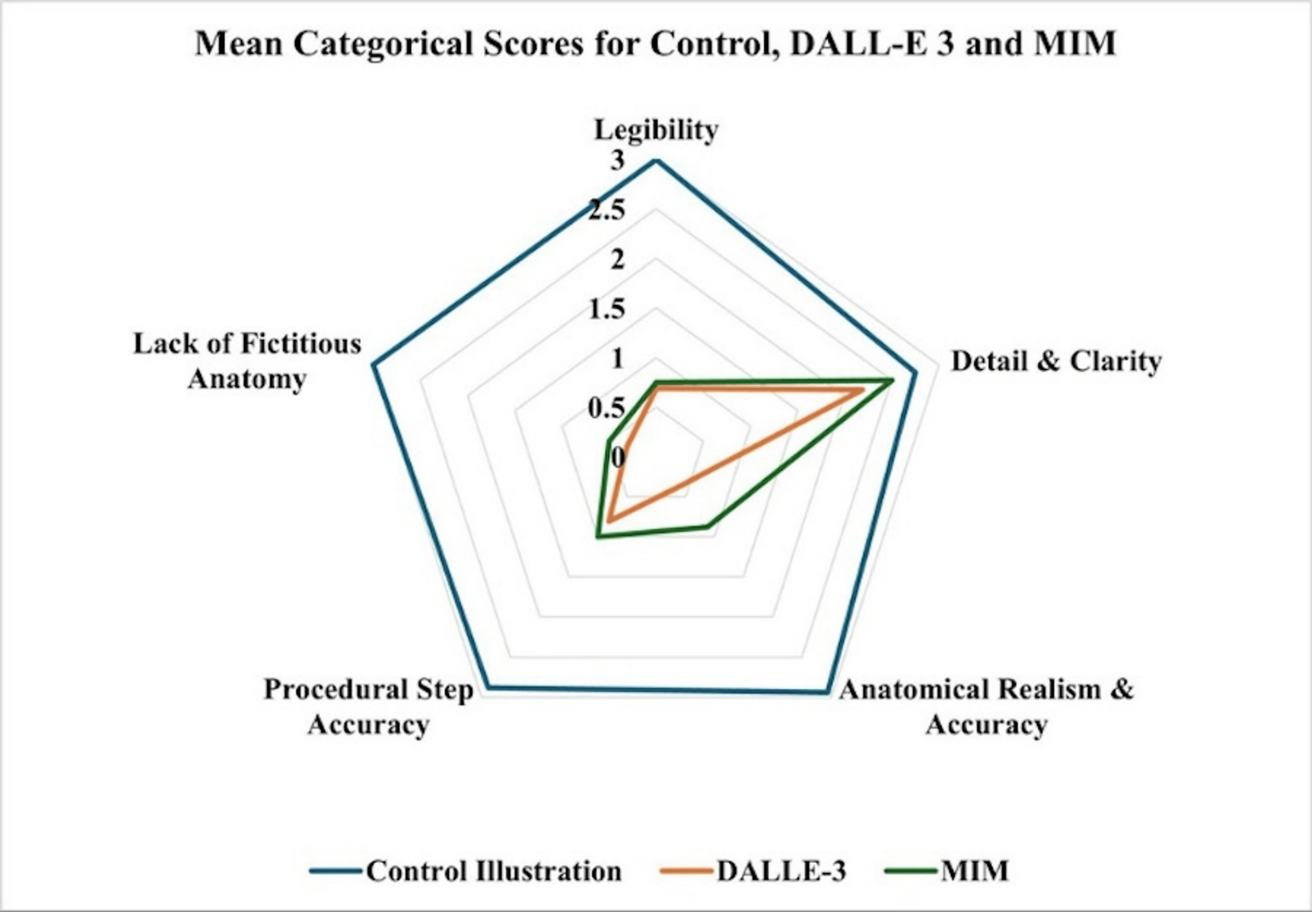
Radial diagram of mean categorical scores of the control and AI generative models Values represented as raw scores (maximum=3 points) AI: artificial intelligence; DALL-E 3: Decoder-Only Autoregressive Language and Image Synthesis 3; MIM: Medical Illustration Master

With regard to legibility, the control illustrations received a significantly higher score than the images produced by DALL-E 3 and MIM (3.00±0.00 vs. 0.69±0.13 and 0.75±0.00; p<0.001) (Table 2, Figure 7). There were no significant differences in legibility scores between DALL-E 3 and MIM (0.69±0.13 vs. 0.75±0.00; p=0.469). Images from all three groups had excellent detail and clarity, with the control illustration group, DALL-E 3, and MIM receiving comparable scores of 2.75±0.29, 2.19±0.24, and 2.50±0.29, respectively (p=0.0504). For the anatomical realism and accuracy category, the control illustration group scored higher than DALL-E 3 and MIM (2.94±0.13 vs. 0.38±0.14 and 0.88±0.25; p<0.001). Additionally, MIM scored higher in anatomical realism and accuracy than DALL-E 3 (0.88±0.25 vs. 0.38±0.14; p=0.009). For the procedural step accuracy category, the control illustration group scored higher than DALL-E 3 and MIM (2.88±0.14 vs. 0.81±0.13 and 1.00±0.20; p<0.001). There were no significant differences in procedural step accuracy scores between DALL-E 3 and MIM (0.81±0.13 vs. 1.00±0.20; p<0.278). For the lack of fictitious anatomy category, the control illustration group scored higher than DALL-E 3 and MIM (3.00±0.00 vs. 0.31±0.24 and 0.50±0.20; p<0.001). There were no significant differences in the lack of fictitious anatomy scores between DALL-E 3 and MIM (0.31±0.24 and 0.50±0.20; p=0.353).

**Table 2:** Individual category scores of control, DALL-E 3, and MIM Values represented as raw scores (maximum=3 points). Values represented as mean±standard deviation. Statistical significance set at p<0.05 *Control illustration score significantly higher than DALL-E 3 and MIM scores **MIM score significantly higher than DALL-E 3 score (p=0.009) DALL-E 3: Decoder-Only Autoregressive Language and Image Synthesis 3; MIM: Medical Illustration Master

	Control illustration	DALLE-3	MIM	P
Legibility	3.00±0.00	0.69±0.13	0.75±0.00	<0.001*
Detail and clarity	2.75±0.29	2.19±0.24	2.50±0.29	0.05048
Anatomical realism and accuracy	2.94±0.13	0.38±0.14	0.88±0.25**	<0.001*
Procedural step accuracy	2.88±0.14	0.81±0.13	1.00±0.20	<0.001*
Lack of fictitious anatomy	3.00±0.00	0.31±0.24	0.50±0.20	<0.001*

Comparison of mean cumulative scores for each transplant procedure

The maximum score in this section is 15 points.

**Figure 8:**
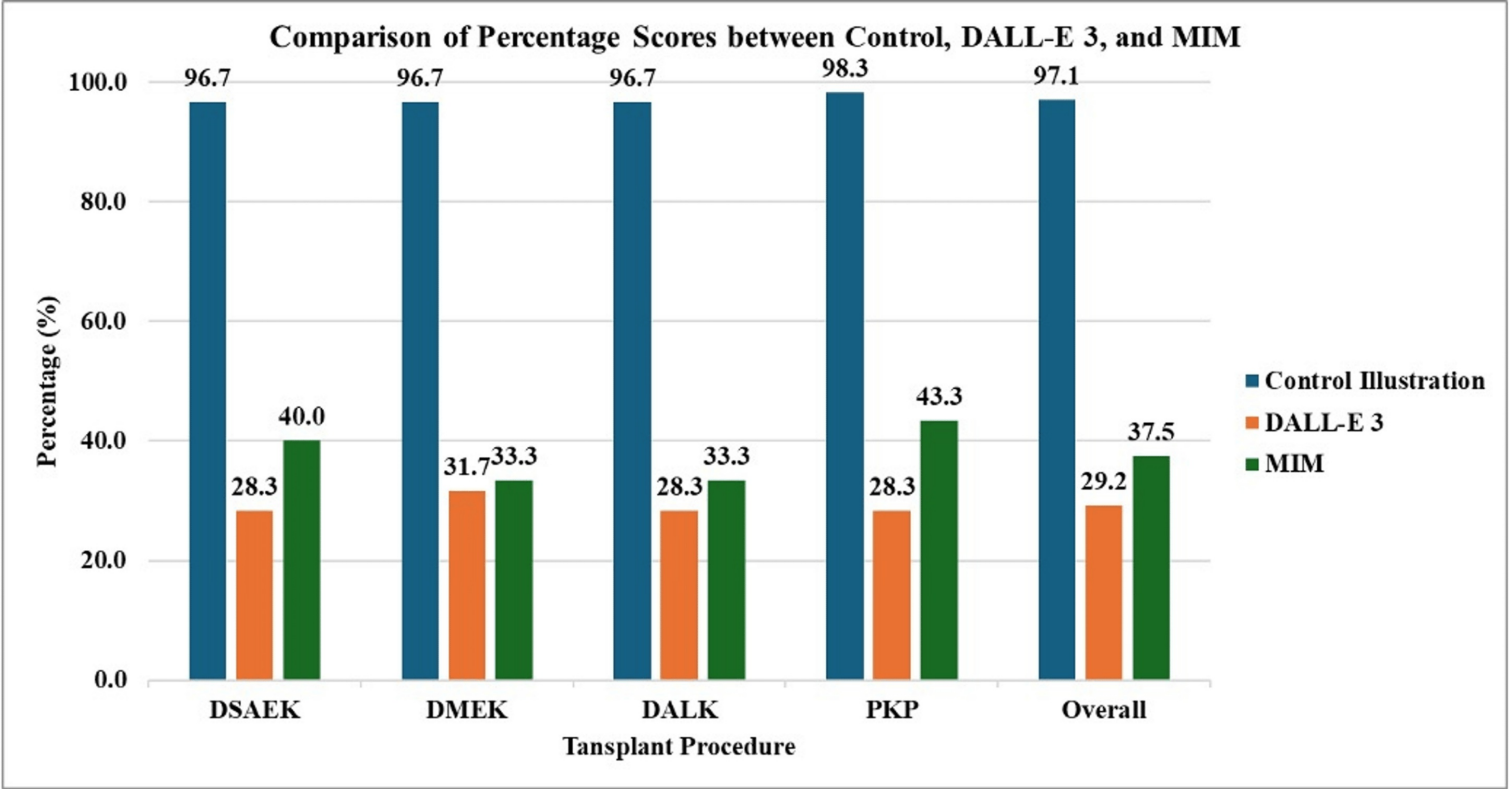
Comparisons of mean cumulative score percentages (%) of the control and generative AI models Values represented as percentages (%) AI: artificial intelligence; DALL-E 3: Decoder-Only Autoregressive Language and Image Synthesis 3; MIM: Medical Illustration Master; DSAEK: Descemet's stripping automated endothelial keratoplasty; DMEK: Descemet's membrane endothelial keratoplasty; DALK: deep anterior lamellar keratoplasty; PKP: penetrating keratoplasty

For DSAEK, the mean cumulative scores of the control illustration group, DALL-E 3, and MIM were 14.5±0.58 (96.7%), 4.25±0.5 (28.3%), and 6.00±2.16 (40%), respectively (Table 3, Figure 8). The control illustration group scored significantly higher than DALL-E 3 and MIM (p<0.001). There were no significant differences in mean cumulative scores between DALL-E 3 and MIM for the DSAEK image (4.25±0.5 vs. 6.00±2.16; p=0.202).

**Table 3:** Mean cumulative scores of control, DALL-E 3, and MIM Values represented as raw scores (maximum=15 points). Values represented as mean±standard deviation. Statistical significance set at p<0.05 *Control illustration score significantly higher than DALL-E 3 and MIM scores DALL-E 3: Decoder-Only Autoregressive Language and Image Synthesis 3; MIM: Medical Illustration Master; DSAEK: Descemet's stripping automated endothelial keratoplasty; DMEK: Descemet's membrane endothelial keratoplasty; DALK: deep anterior lamellar keratoplasty; PKP: penetrating keratoplasty

	Control illustration	DALL-E 3	MIM	P*
DSAEK	14.5±0.58	4.25±0.5	6.00±2.16	<0.001
DMEK	14.5±0.58	4.75±1.29	5.00±1.15	<0.001
DALK	14.5±0.58	4.25±0.98	5.00±1.63	<0.001
PKP	14.75±0.50	4.25±2.06	6.50±2.38	<0.001
Overall	14.56±0.51	4.38±1.2	5.63±1.82	<0.001

For DMEK, the mean cumulative scores of the control illustration group, DALL-E 3, and MIM were 14.5±0.58 (96.7%), 4.75±1.29 (31.7%), and 5.00±1.15 (33.3%), respectively. The control illustration group scored significantly higher than DALL-E 3 and MIM (p<0.001). There were no significant differences in mean cumulative score between DALL-E 3 and MIM for the DMEK image (4.75±1.2 vs. 5.00±1.15; p=0.786).

For DALK, the mean cumulative scores of the control illustration group, DALL-E 3, and MIM were 14.5±0.58 (96.7%), 4.25±0.98 (28.3%), and 5.00±1.63 (33.3%), respectively. The control illustration group scored significantly higher than DALL-E 3 and MIM (p<0.001). There were no significant differences in mean cumulative score between DALL-E 3 and MIM for the DALK image (4.25±0.98 vs. 5.00±1.63; p=0.637).

For PKP, the mean cumulative scores of the control illustration group, DALL-E 3, and MIM were 14.75±0.50 (98.3%), 4.25±2.06 (28.3%), and 6.50±2.38 (43.3%), respectively. The control illustration group scored significantly higher than DALL-E 3 and MIM (p<0.001). There were no significant differences in mean cumulative score between DALL-E 3 and MIM for the PKP image (4.25±2.06 vs. 6.50±2.38; p=0.247).

With regard to the overall mean cumulative score, the control illustration group, DALL-E 3, and MIM achieved a score of 14.56±0.51 (97.1%), 4.38±1.2 (29.2%), and 5.63±1.82 (37.5%), respectively. The control illustration group significantly scored higher than DALL-E 3 and MIM (p<0.001). However, MIM also scored significantly higher than DALL-E 3 (5.63±1.82 vs. 4.38±1.2; p=0.017).

Comparison of grading performance between the reviewers and ChatGPT-4o

The maximum score in this section is 15 points.

**Figure 9:**
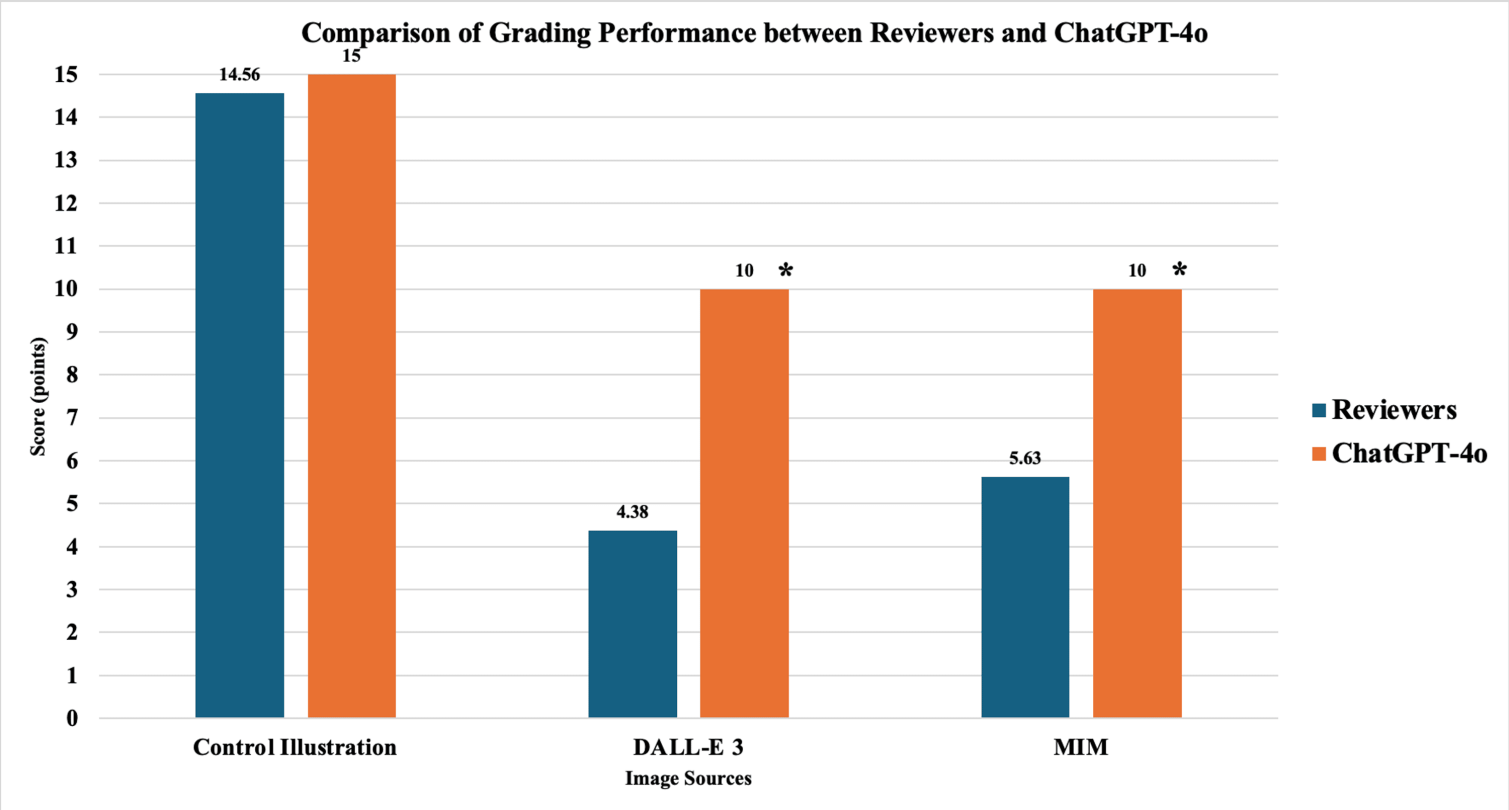
Grading performance comparisons between reviewers and ChatGPT-4o Values represented as raw scores (maximum=15 points). Statistical significance set at p<0.05 *ChatGPT-4o score significantly higher than reviewer score (p<0.001) DALL-E 3: Decoder-Only Autoregressive Language and Image Synthesis 3; MIM: Medical Illustration Master

For the DALL-E 3-produced images, the mean score for the four transplant procedures was significantly lower from the reviewers compared to the mean score given by ChatGPT-4o (4.38±1.20 (29.2%) vs. 10.0±0.0 (66.6%); p<0.001) (Figure 9). For the MIM-produced images, the mean score for the four transplant procedures was significantly lower from the reviewers compared to the mean score given by ChatGPT-4o (5.63±1.82 (37.5%) vs. 10.0±0.0 (66.6%); p<0.001). However, for the control illustrations initially created by the authors, the mean score for the four transplant procedures was not significantly different between the reviewers and ChatGPT-4o (14.56±0.13 (97.1%) vs. 15.0±0.0 (100%); p>0.05).

## Discussion

There is limited exploration of AI's potential in creating medically accurate illustrations, especially in the field of ophthalmology. A previous study attempted to generate novel illustrations of Horner syndrome and hypothyroidism using AI models, while other studies explored AI's capability in image generation within radiology [10-12]. This study investigates the accuracy of the DALL-E 3 and MIM AI models in generating medical illustrations specifically for several corneal transplant surgeries. Findings revealed significant inaccuracies in the AI-generated images compared to current established resources freely available online.

Although both generative AI models in this study produced images with excellent clarity and detail, they exhibited severe limitations in anatomical and procedural step accuracy. Similar to our study, Noel prompted DALL-E 2 to create detailed illustrations of the human skull, heart, and brain. They found that although the images were aesthetically pleasing and of high quality, they omitted crucial anatomical features such as the mental and supraorbital foramina of the skull, the origins of the coronary arteries in the heart, and the locations of gyri and sulci in the brain [13]. Both models in our study additionally created fictitious and excessive anatomical structures related to the prompted procedure as seen in Figure 4A, placing them in erroneous areas. Adams et al. similarly described that when prompting DALL-E-2 to create radiological illustrations, the generative AI model would create extra fingers or place foreign bodies resembling a prosthesis in the produced images [11]. It is evident from our results and those of the aforementioned studies that generative AI models such as DALL-E and MIM have the capability of creating artistic designs of excellent image quality, but fail at depicting accurate anatomical structures. 

Our study additionally shows that when asking ChatGPT-4o to grade the fictitious AI-generated images based on our own created grading system, it artificially inflated its scores, scoring it much higher than our independent reviewers. However, scores between ChatGPT-4o and our reviewers were not significantly different when it came to the control illustration images created by the authors. Although, to the best of our knowledge, there are no studies in the current literature describing AI's assessment of its own generated imagery, one study by Azad et al. demonstrated that human graders scored computer science questions completed by students as 51% correct, compared to 89% by AI graders, with a 12% false-positive rate [14]. A plausible explanation for ChatGPT-4o's inflated scores for the AI-generated images in our study is that the AI's reasoning and interpretation were biased, as the images were produced by its own system. 

Despite utilizing a subscription-based AI service, we observed several shortcomings in our study. The prompts administered (Table 1) were created by clinicians rather than professional prompt engineers, which may have impacted the quality and specificity of the generated images. Additionally, the algorithms used by the AI models are not fully transparent to us, making it difficult to verify the sources and processes through which AI generates these images. There are also concerns regarding reproducibility, as each prompt input may yield a different unique image. We noted that AI systems are inept in the critical evaluation of their produced images. There is a lack of a "self-feedback" mechanism resulting in amplified inconsistencies in the majority of the produced illustrations, insinuating that current AI large language models are unable to translate text to image creation. Some may also argue that the statistical power of 0.30 was weak due to our small sample size. However, our power analysis was set at an ⍺ of 0.05, allowing us to reject our null hypothesis despite our small sample size. Furthermore, we did not assess or exhaust alternative AI image generation models such as Midjourney or Stable Diffusion. However, we selected DALL-E 3 due to its reputation as a well-known image recognition software. While this decision could be seen as a limitation, we believed it was important to use a widely recognized and validated platform to establish a baseline for future comparisons. Our findings highlight the need for further research using a broader range of AI tools to fully understand their capabilities and limitations in medical illustration.

## Conclusions

This study emphasizes the current inadequacies of AI models in generating medically accurate illustrations as it specifically relates to corneal transplant procedures such as DSAEK, DMEK, DALK, and PKP. While the tested AI models prevail in their image-to-text interpretations and creation of vibrant artwork, we found no educational value in the produced images. 

It is crucial to approach AI-generated images and videos with skepticism. The public should exercise caution and vigilance to identify false or inaccurate information to mitigate its risks. While AI has the potential to revolutionize many aspects of medical practice, especially in ophthalmology, our findings underscore the need for further development before it can be relied upon for professional medical illustration. Continued investigation is paramount in ensuring AI's utility as a reliable and valuable tool for medical learning and education.

## Figures and Tables

**Figure 10:**
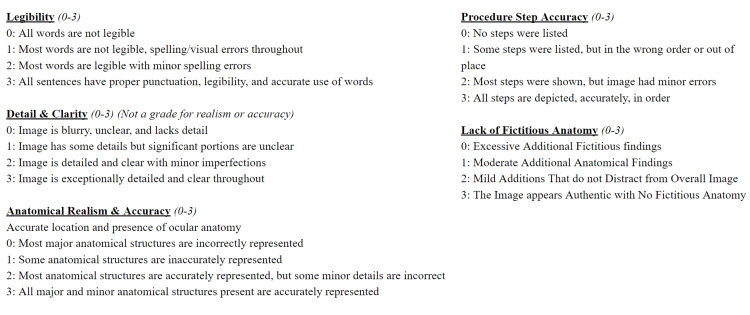
Objective grading system used to assess each resultant AI-generated image and control illustration Image created by the authors with Microsoft Office AI: artificial intelligence
